# Local biases drive, but do not determine, the perception of illusory trajectories

**DOI:** 10.1038/s41598-020-64837-0

**Published:** 2020-05-08

**Authors:** Tamara N. Gheorghes, Paul Richardson, John Reidy

**Affiliations:** 1 University of Leicester, Leicester, England; 2 Sheffield Hallam University, Sheffield, England

**Keywords:** Psychology, Human behaviour, Neuroscience, Visual system, Motion detection

## Abstract

When a dot moves horizontally across a set of tilted lines of alternating orientations, the dot appears to be moving up and down along its trajectory. This perceptual phenomenon, known as the slalom illusion, reveals a mismatch between the veridical motion signals and the subjective percept of the motion trajectory, which has not been comprehensively explained. In the present study, we investigated the empirical boundaries of the slalom illusion using psychophysical methods. The phenomenon was found to occur both under conditions of smooth pursuit eye movements and constant fixation, and to be consistently amplified by intermittently occluding the dot trajectory. When the motion direction of the dot was not constant, however, the stimulus display did not elicit the expected illusory percept. These findings confirm that a local bias towards perpendicularity at the intersection points between the dot trajectory and the tilted lines cause the illusion, but also highlight that higher-level cortical processes are involved in interpreting and amplifying the biased local motion signals into a global illusion of trajectory perception.

## Introduction

Accurately perceiving the trajectory of a moving object is important, as it helps with anticipating its future position (e.g., avoiding a potential danger), interacting with the object (e.g., catching a ball), and identifying the object (e.g., the flight of a bird). Throughout everyday activities, the human visual system tends to perform well at this task, but in specific situations it may produce a visual illusion. Such illusions are not mere curiosities – they expose the underlying mechanisms of visual processing.

**Figure 1:**
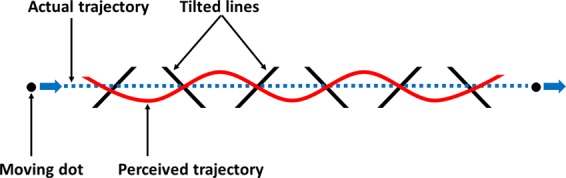
Schematic representation of the slalom illusion. The moving dot follows a straight trajectory, but due to its intersection with the tilted lines, the perceived trajectory is sinusoidal.

Cesàro and Agostini^1^ have described the *slalom illusion*, whereby the straight horizontal trajectory of a dot appears to be undulating when moving across a sequence of alternating tilted inducer lines (Fig. 1). Specifically, the perceived trajectory of the dot appears to bend so as to intersect the lines at a perpendicular angle. The slalom illusion has received little attention in the literature since its initial publication, but it does offer important insight into the neurocognitive mechanisms that allow observers to quickly construct a coherent trajectory percept. In their original paper, the authors reported three main findings: more acute angles of intersection, slower dot speeds, and smaller distances between the lines all caused a larger illusory amplitude to the perceived trajectory. To explain the slalom illusion, Cesàro and Agostini proposed that when the dot is near the inducer lines, the perception of the dot motion is locally biased or distorted towards a motion path perpendicular to the inducer lines. In an attempt to achieve a coherent percept, the visual system, as a compromise with the veridical horizontal trajectory, arrives at a smooth but sinusoidal trajectory percept.

The unique characteristics of the slalom illusion are that it both pertains to a *single moving object* following a *trajectory* of illusory shape, and is present in *foveal vision*. However, other illusions exist which share part of the slalom illusion’s phenomenology and, potentially, of its underlying mechanisms. Indeed, illusory normalisation towards perpendicular angles has been observed in a wide range of visual displays, both static and kinetic.

First, a number of classical geometric illusions feature static lines that in reality are straight or parallel, but appear to be bent^2,3^ or at an angle with each other^4,5^. In either case, the distortion tends towards perpendicular angles of intersection. Although the original versions were static in nature, kinetic versions of these illusions have also been reported in the literature^6–9^. The perpendicularity bias has often been proposed to be rooted in lateral inhibition of orientation-selective neurons, and as a consequence, lines intersecting at acute angles appear to repel each other towards a more perpendicular angle^10^. In another view, the perpendicularity bias is a result of Bayesian inference^11–14^, based on the fact that perpendicular angles are more likely to occur in the visual environment (for instance, trees on the horizon). Lastly, in the differential processing account^15,16^, a perpendicularity bias observed with random dot kinematograms in the context of a single tilted line was successfully modelled on the psychophysical finding that object-relative motion components (perpendicular to the line) contribute more to the perceived dot speed than non-object-relative motion components (parallel to the line)^17^.

Second, a number of more recent illusions do pertain to single-object trajectory motion, but only when the reliability of visual information is low. Anstis^18–20^ reported the Furrow Illusion in peripheral vision, whereby a dot moving vertically up and down across a background grating of tilted high-contrast stripes appeared to follow along the orientation of the grating instead. The authors proposed that peripheral vision confounds the orientation signals of the background with the motion direction of the dot. Similarly, in the Squirming illusion^21^, a line segment moving straight across a zigzag pattern of tilted lines in peripheral vision appeared to follow a curved trajectory along the pattern instead. That is, unlike the slalom Illusion, the perceived phase of the sinusoidal modulation followed the orientation of the inducing lines instead of being perpendicular to it. However, the authors also reported that in central vision the observed pattern was indeed that of the slalom illusion: the phase of the trajectory was in opposition to that of the inducing lines. A similar observation was made in the case of the Bicycle illusion^22^; at a small display and low dot contrast, the dot trajectory appeared to move in phase with a sinusoidal static set of lines, whereas at a large display size, an opposite phase was perceived. The authors suggested that the poor reliability of visual information on dot position caused this observation, reminiscent of the Furrow Illusion. Indeed, it could be argued that viewing a display in peripheral vision has much the same effect as using a small display size, due to the increased receptive field sizes at greater retinal eccentricities.

The current study seeks to explore the empirical boundaries of the slalom illusion phenomenon, and to further test the high-level theoretical proposition put forward by Cesàro and Agostini.

In Experiment 1, Cesàro and Agostini’s observation that smaller distances between the inducing lines led to a stronger illusion will, through an occlusion manipulation, be experimentally disentangled into two possible drivers: the distance itself, or the availability of the trajectory information away from the points of intersection. In the ‘perceptual compromise’ view of Cesàro and Agostini, the availability should be the driver.

In Experiment 2, the observation that higher dot speeds led to a reduced illusion will also be disentangled into two possible drivers: the increased salience of the veridical horizontal motion in a bottom-up, passive view on the perceptual compromise, or the decreased opportunity to construe a compromise trajectory shape at a higher level of processing. The crucial manipulation used was that of the dot speed during occlusion.

In Experiment 3, the slalom display was inverted, using a veridically sinusoidal dot trajectory with vertical inducer lines. If the perpendicularity bias is the sole driver of the slalom illusion, a reduced perceived amplitude of the dot trajectory would be expected.

Finally, in Experiment 4, to elucidate the role of smooth pursuit eye movements in the slalom illusion, we tested whether the illusion still occurred under conditions of constant fixation. In addition, we systematically tested whether retinal eccentricity affected the amplitude of the slalom illusion, and whether indeed it was replaced by the Squirming or Bicycle illusion as the reliability of positional information decreased with increasing eccentricity.

## Results

### Experiment 1 – Partial occlusion of the trajectory

A central feature of the theoretical view of Cesàro and Agostini^1^ is the idea that the perceived sinusoidal trajectory of the slalom illusion is a compromise between the veridical horizontal motion on the one hand, and the local distortions towards perpendicular angles on the other hand. Indeed, there is ubiquitous evidence of such spatio-temporal integration of motion vectors, across an area of up to one degree of visual angle and a time window of 100 ms^23–30^. In support of their view, the authors particularly emphasised their finding that smaller distances between the inducing lines resulted in a smaller illusory amplitude to the trajectory; because less non-distorted motion was available between the inducing lines, the compromise percept was more strongly biased towards a sinusoidal trajectory.

**Figure 2:**
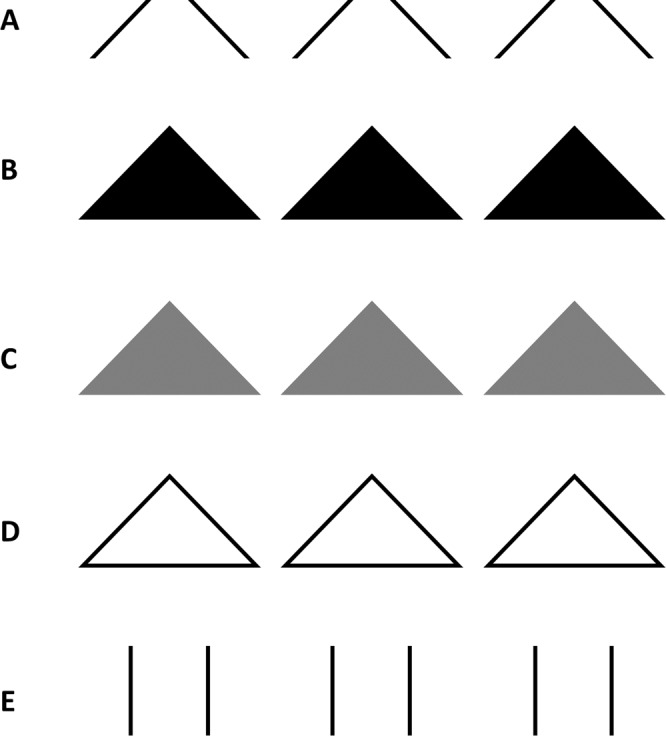
Illustration of the five experimental conditions of Experiment 1: *original slalom* (**A**), *occluding triangles* (**B**), *grey triangles* (**C**), *transparent triangles* (**D**), and *control* (**E**).

To further test this proposition, we partially occluded the trajectory instead of shortening it, expecting a qualitatively similar result of increased illusory amplitude. The manipulation of partial occlusion was achieved by filling up the space between the tilted lines as triangles. As an additional control for the confounding introduction of the horizontal line which formed the base of the triangles in the display, non-occluding triangle conditions were added to the experimental design. Figure 2 illustrates the five conditions: control condition with vertical lines (Fig. 2E), original slalom condition with tilted lines (Fig. 2A), occluding triangles condition with black triangles (Fig. 2B), and two non-occluding triangle conditions, grey triangles (Fig. 2C) and transparent triangles (Fig. 2D). The dependent variable was measured as a subjective assessment of the sinusoidal amplitude.

**Figure 3:**
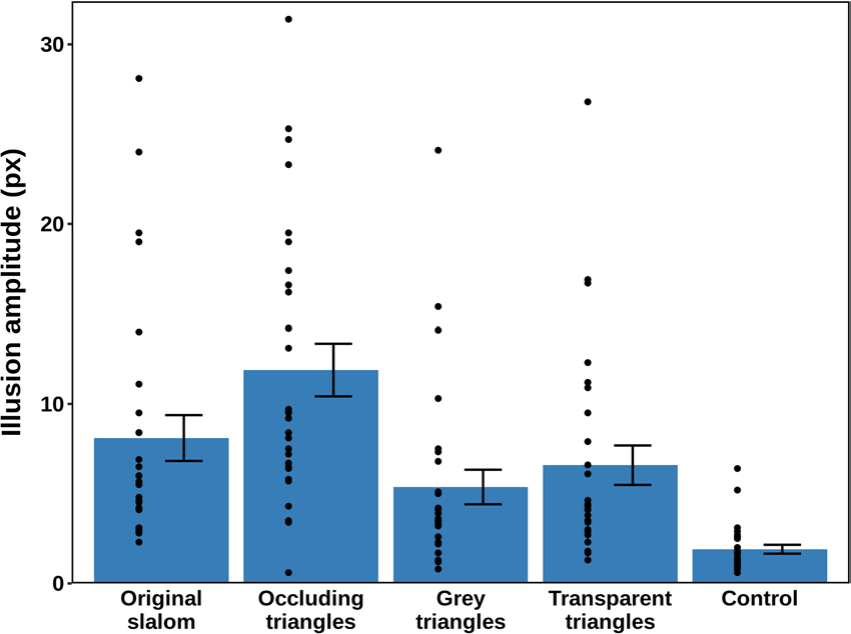
Mean amplitudes and standard errors for the five experimental conditions in Experiment 1. The black dots represent the condition means for the individual participants retained in the analysis (N = 28).

An ANOVA with Greenhouse-Geisser correction was performed on the log-transformed data, of which the conditional means are shown in Fig. 3. The background conditions had a significant effect on the illusion amplitude [*F* (2.99, 80.73) = 86.12, *p* < 0.001, η_p_^2^ = 0.761]. The statistical power based on this η_p_^2^ was estimated at >0.999.

Bonferroni-corrected post-hoc pairwise comparisons showed that the illusion amplitude was significantly larger in the occluding triangles condition compared with all other conditions: original slalom (*p* = 0.006), grey triangles, transparent triangles, and control (all *p* < 0.001). In the control condition, the magnitude of the illusion was significantly smaller than in all the other conditions (all *p* < 0.001). In the original slalom condition, the illusion amplitude was significantly larger when compared to the grey triangles (*p* = 0.001) and transparent triangles (*p* < 0.001). There was no difference between the grey triangles and transparent triangles conditions (*p* = 0.187). The control condition itself was non-zero, but this was expected because of response error in adjusting the probe line.

In conclusion, the slalom illusion was replicated and, as hypothesised, its magnitude increased further when the trajectory was intermittently occluded with black triangles. The slalom effect was reduced, but still present, in both non-occluding triangles. It can be speculated that the presence of the base of the triangle provided a more useful frame of reference for the estimation of the amplitude of the trajectory than the tilted lines of the original slalom display. More importantly, occlusion rather than triangular shape was the necessary condition for the amplification of the original slalom effect.

### Experiment 2 – ISI of occlusion

Cesàro and Agostini^1^ observed that higher dot speeds reduced the magnitude of the slalom illusion. Two different explanations can be offered for this finding. First, the fast horizontal motion signal might simply be more salient, giving more bottom-up weight to the veridical trajectory in the perceptual compromise and therefore reducing the illusory amplitude. Second, the faster pass-through time of the dot through the display might be harder to reconcile with a longer sinusoidal trajectory than with a straighter trajectory. That is, it is easier to see a slowly moving dot as moving along a longer trajectory than a fast moving dot. Comparable effects have been observed in the literature on apparent motion^31,32^, whereby the illusory path of motion can be perceived as being curved^33^ only if the inter-stimulus interval (ISI) between the successive dot presentations is sufficiently long^34^ and consistent with the perceived cause of the motion^35^. It has previously been suggested that apparent motion and continuous trajectory perception have a shared neuronal basis^36,37^.

**Figure 4:**
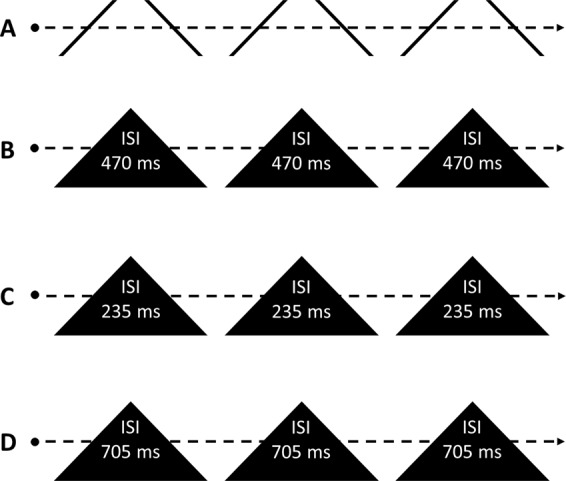
Illustration of the four experimental conditions in Experiment 2: *original slalom* (**A**), *original ISI* (**B**), *short ISI* (**C**), and *long ISI* (**D**). In all conditions, the dot is moving at the same speed (5 cm/s) on the visible parts of the trajectory, whereas in the occluded parts of the trajectory the speed varied according to the ISI.

We tested the predictions of both hypotheses against each other by building on the occlusion manipulation of Experiment 1. That is, the ISI was manipulated by speeding up or slowing down the dot as it invisibly traversed behind the triangular occluders. The *visible* motion vectors remained the same. The experimental conditions are illustrated in Fig. 4. Three different ISIs of occlusion were used: 470 ms (original ISI; corresponding to the non-occluded speed; Fig. 4B), 235 ms (short ISI; Fig. 4C), and 705 ms (long ISI; Fig. 4D). In addition, a fourth condition was included which corresponded to the original slalom illusion, without any occlusion and with a visible dot speed equal to the original ISI condition (Fig. 4A). If the salience of faster dot motion led to the reduced illusion magnitude reported by Cesàro and Agostini, ISI manipulation should not matter. If the congruence of pass-through time and trajectory amplitude drove the effect, ISI should be inversely related to the magnitude of the illusion.

**Figure 5:**
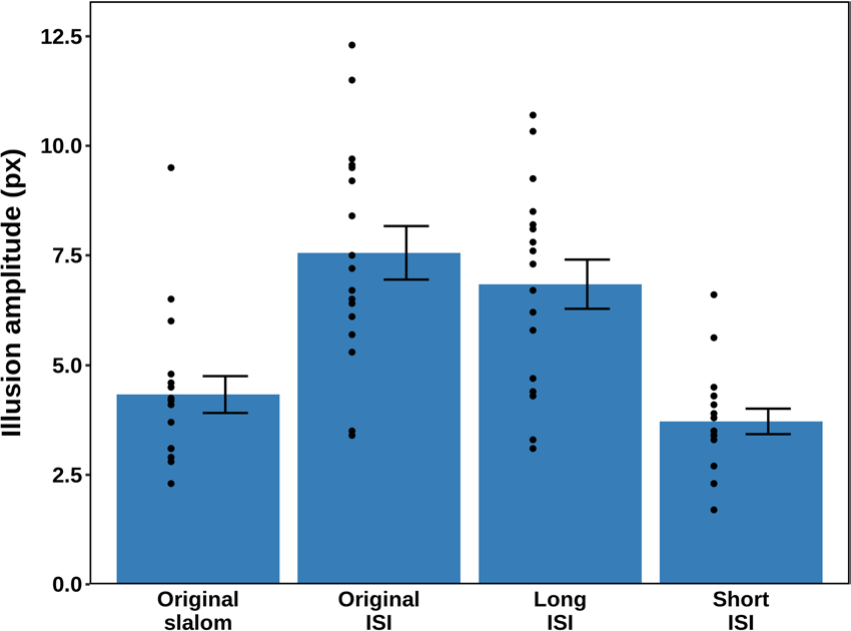
Mean amplitudes and standard errors for the four experimental conditions in Experiment 2. The black dots represent the condition means for the individual participants (N = 17). There were no outliers.

The conditional means of the data are shown in Fig. 5 and were analysed using repeated-measures ANOVA with Greenhouse-Geisser correction. The background manipulation had a significant effect on the perception of the dot trajectory [*F* (2.04, 32.65) = 44.80, *p* < 0.001, η_p_^2^ = 0.737]. The statistical power based on this η_p_^2^ was estimated at > 0.999.

Bonferroni-corrected post-hoc pairwise comparisons showed that the reported illusion amplitude was significantly larger in the original ISI condition compared with the original slalom condition (p < 0.001) and short ISI condition (p < 0.001). Also, the illusion amplitude was significantly larger in the long ISI condition compared with the original slalom condition (p < 0.001) and short ISI condition (p < 0.001). There was no difference in the illusion amplitude between the original ISI and the long ISI conditions (p = 0.336) or between the original slalom and the short ISI conditions (p = 0.091).

In conclusion, the occlusion effect of Experiment 1 was replicated, but the effect of the ISI manipulation was mixed: the long ISI condition did not affect the occluded slalom illusion, whereas the short ISI condition reduced it to the level of the classical, non-occluded slalom illusion. This demonstrates that the dot speed effect reported by Cesàro and Agostini was not caused by the more salient presence of faster visible motion. However, the results only partially agree with the alternate hypothesis, where the illusory trajectory amplitude was inferred partially as a function of its plausible length given the ISI. Possibly, this can be attributed to a ceiling effect, whereby the slalom illusion was already maximal in the baseline original ISI occlusion condition, and could not be increased further by prolonging the ISI.

### Experiment 3 – Inverted slalom illusion

Cesàro and Agostini^1^ found a near-linear decrease in the illusion magnitude as the angle of intersection increased. This finding in particular supported the hypothesis that local distortions in perceived motion direction occurred around the points of intersection between the tilted lines and the dot trajectory, such that the angle of intersection appeared to be biased towards perpendicularity - the rationale being that angles already closer to perpendicularity caused less local distortion. Indeed, a bias in the human visual system towards perpendicular angles has previously been reported using both static and moving lines^10–14,23,38–40^. If indeed the angle of intersection drives the slalom illusion, it should be possible to invert the direction of the illusion by letting a sinusoidal dot trajectory move across a set of vertical line stimuli. The bias towards perpendicularity at the points of intersection should then result in a decreased perceived amplitude, compared to a control condition with the tilted lines of the classic slalom display. In addition, it would be expected that the inverted slalom illusion, similar to Experiment 1, would increase in strength (that is, a smaller trajectory amplitude would be perceived) when the trajectory is partially occluded, because less veridical motion information is available to counteract the perpendicularity bias.

**Figure 6:**
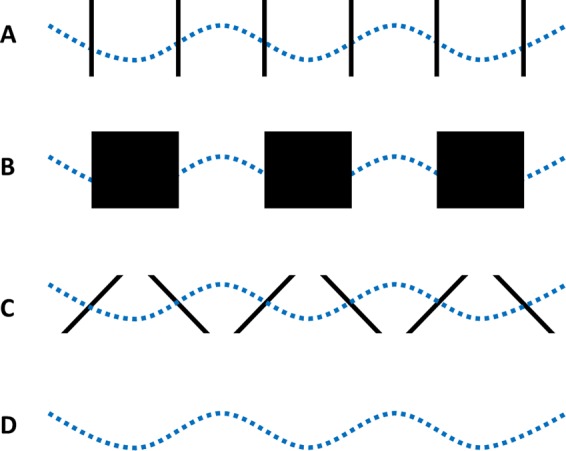
Illustration of the four experimental conditions in Experiment 3: *inverted slalom* (**A**), *inverted occluded* (**B**), *inverted control* (**C**), and *blank* (**D**). The dotted line represents the veridical sinusoidal trajectory of the moving dot.

The stimulus conditions are shown in Fig. 6. In the Blank condition (Fig. 6D), no background lines were shown. The veridical trajectory was sinusoidal, with an amplitude of 10 pixels. The inverted control condition (Fig. 6C) contained tilted lines, whereas the inverted slalom condition (Fig. 6A) had a background of vertical lines. In the inverted occluded condition (Fig. 6B), dark squares occluded half of the space between the vertical lines.

**Figure 7:**
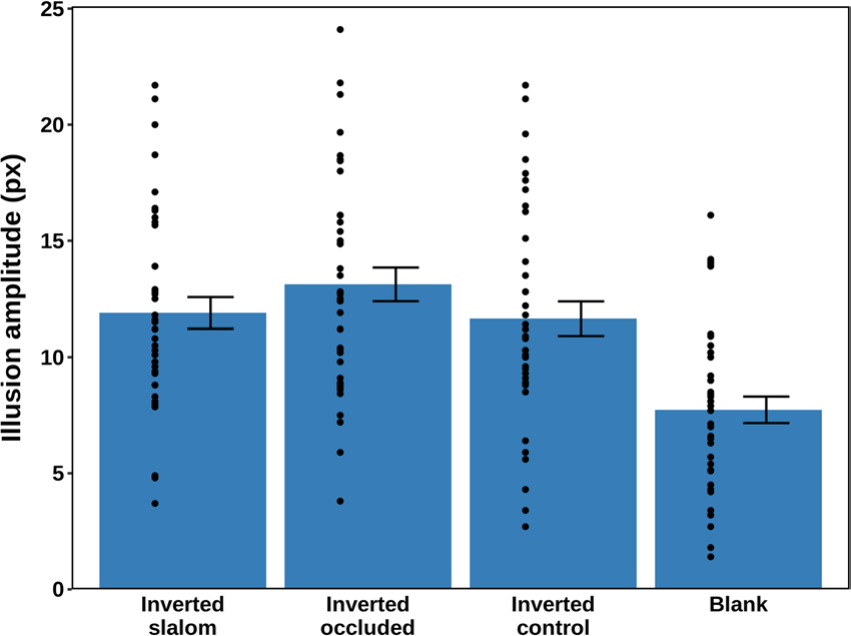
The mean amplitudes and standard errors for the four experimental conditions in Experiment 3. The black dots represent the condition means for the individual participants (N = 40). There were no outliers.

The conditional means are shown in Fig. 7, and the data were analysed using repeated-measures ANOVA with Greenhouse-Geisser correction. The background conditions had a significant effect on the perception of the dot trajectory [*F* (2.44, 95.20) = 50.99, *p* < 0.001, η_p_^2^ = 0.567]. The statistical power based on this η_p_^2^ was estimated at > 0.999.

Bonferroni-corrected post-hoc pairwise comparisons showed that in the inverted occluded condition, the amplitude was significantly higher than in all three other conditions: inverted slalom (*p* = 0.002), inverted control (*p* = 0.043), and Blank (*p* < 0.001). The amplitude reported for the Blank condition was significantly lower than for inverted slalom (*p* < 0.001) and inverted control (*p* < 0.001). No significant difference was found between the inverted slalom condition and the inverted control condition (*p* > 0.999).

In order to investigate whether the perceived amplitudes in each of the conditions were overestimated or underestimated relative to the real amplitude of the trajectory of 10 pixels, four one-sample two-tailed t-tests were performed. Bonferroni corrections were applied and the results showed that in the inverted slalom (*p* = 0.0315) and the inverted occluded (*p* < 0.001) conditions, the amplitude of the trajectory was significantly overestimated by participants, whereas in the Blank condition it was significantly underestimated (*p* = 001). In the inverted control condition, no significant difference with regard to the real amplitude of the trajectory was observed after Bonferroni correction (*p* = 0.125).

To investigate whether the underestimation of the illusion amplitude in the Blank condition occurred systematically, or whether it indicated a general lack of accuracy in perceiving the trajectory in this condition, we performed an additional experiment reported in Supplementary Information, available online. The results showed that the proportional underestimation was similar across different trajectory amplitudes, although significantly larger for the smallest amplitude. The underestimation of amplitudes in the Blank condition can potentially be viewed in the light of the differential processing view on dot motion^15,16^, whereby sensitivity to non-object relative velocities is decreased.

To summarise, the results show that there is no difference in perceived trajectory amplitude, when comparing the vertical line inducers condition with the tilted line inducers condition. The hypothesis that the angle of intersection and the bias towards perpendicularity are the only drivers of the slalom illusion can therefore not be supported. Whereas the original illusory effect with a straight motion path can be reproduced reliably in a variety of operationalisations of the classic slalom illusion, it cannot simply be inverted to result in a decreased perceived trajectory amplitude.

### Experiment 4 – Effects of constant fixation and retinal eccentricity

In the previous experiments, participants were instructed to follow the dot with their gaze. This was in line with the methods of Cesàro and Agostini^1^. This implies that eye movements largely stabilised the moving dot on the retina. It is therefore a valid empirical question to ask whether the slalom illusion would also occur under conditions of constant fixation, when the projection of the dot does move across the retina. If it does not, the explanatory mechanism should be sought in processes related to eye movements rather than motion perception alone. In the current experiment, an eye tracker was used to ensure participants maintained their gaze at a fixation dot.

A related question is whether the classical slalom illusion, if it occurs at all with a fixational viewing strategy, is affected by retinal eccentricity - the distance from the projection of the stimulus on the retina to the fovea centralis. Although sensitivity to motion is relatively preserved in peripheral vision^41–43^, impaired coding of position and orientation^44–50^ and confounding of line orientation and motion direction^18,19,21^ could affect both the presence and the magnitude of the slalom illusion. Indeed, in the case of the Squirming, Furrow and Bicycle illusions, which occur either in peripheral vision or in small, low-contrast displays, the illusory motion direction was reported to be parallel rather than perpendicular to the inducing background lines. Although on the basis of existing literature it can be suspected that the dot trajectory will be perceived to be in phase with the inducing lines at increasing eccentricities, this has not been systematically tested before.

Participants in the current experiment were asked to report the amplitude of the illusory trajectory in each of the three Eccentricity conditions (Low - Mid - High) combined with the classical slalom effect manipulations (control and tilted lines). In addition, they were asked to indicate explicitly whether they had perceived a slalom, squirming, or straight trajectory.

#### Illusion amplitude

**Figure 8:**
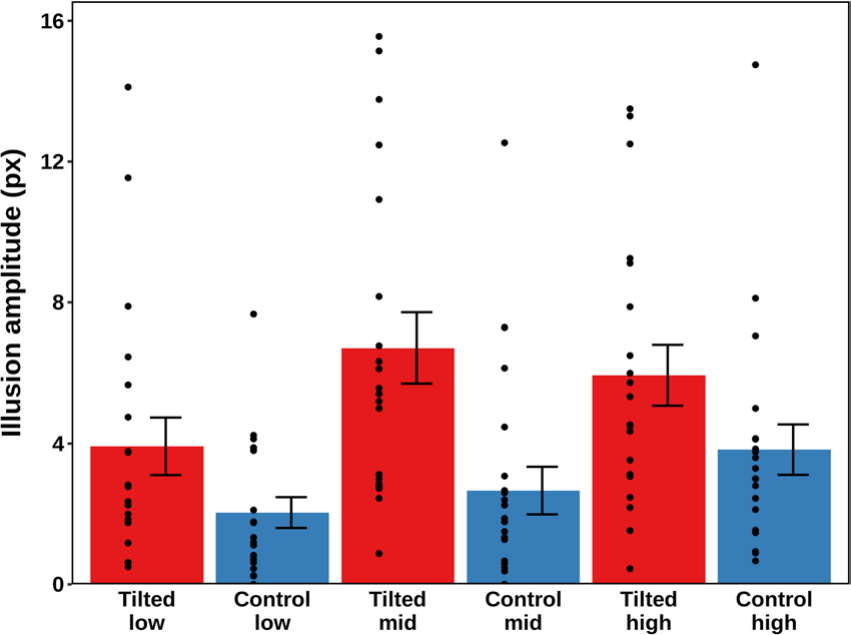
Mean amplitudes and standard errors for the six experimental conditions in Experiment 4. The tilted conditions are represented in blue, whereas the control conditions are represented in red. The black dots represent the condition means for the individual participants (N = 20). There were no outliers.

The effect of the experimental conditions on the perception of the trajectory was analysed using a two-way repeated-measures ANOVA with Greenhouse-Geisser correction where applicable. All the inferential statistics were calculated based on the computed natural logarithms. The conditional means are shown in Fig. 8. Both main effects and their interaction were significant: stimulus type [*F* (1, 19) = 41.12, *p* < 0.001, η_p_^2^ = 0.684], eccentricity [*F* (2, 38) = 19.57, *p* < 0.001, η_p_^2^ = 0.507], and stimulus type by eccentricity [*F* (2, 38) = 5.26, *p* = 0.010, η_p_^2^ = 0.217]. The power estimates based on this η_p_^2^ were > 0.999 for both the main effects and the interaction effect.

In order to investigate the interaction of both eccentricity and stimulus type variables, a simple effects analysis was conducted using 15 post-hoc paired t-tests, to which a Bonferroni correction was applied. Notably, within the tilted conditions, there was a significant difference between the Low and the Mid eccentricities (p = 0.013), but not between the Low and the High or the Mid and the High eccentricities. Within the control conditions, only the difference between the Low and the High eccentricity was significant (p < 0.001). There was a significant difference between the tilted and the control condition for Low (p < 0.04), Mid (p = 0.001) and High (p = 0.002) Eccentricity.

#### Response type

**Figure 9:**
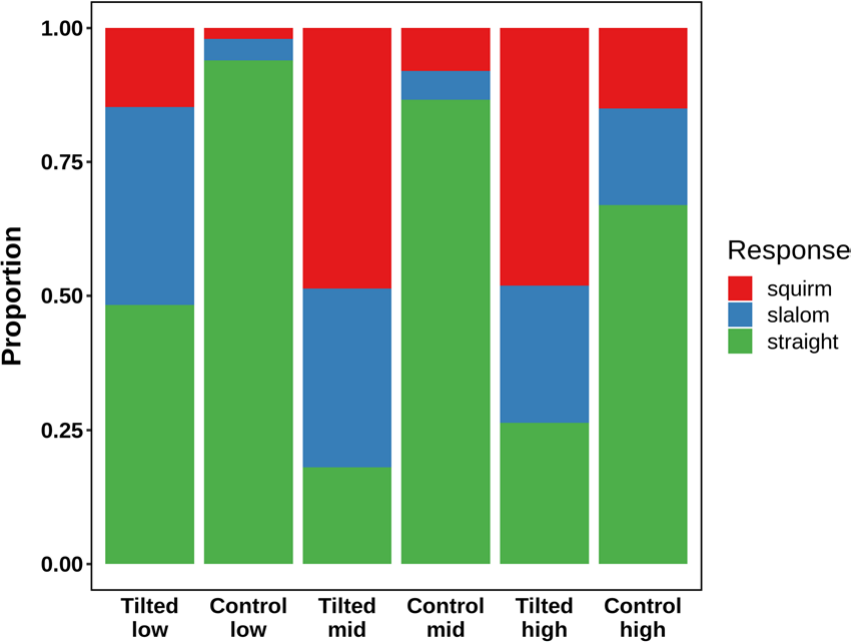
Proportions of the illusion type response for the six experimental conditions in Experiment 4. The stacked bars represent the mean proportion across all participants (N = 20).

Three response options were offered to participants to indicate the type of illusion they had perceived: straight (no illusion), slalom (trajectory intersecting the inducing lines), and squirm (trajectory along the inducing lines). Figure 9 displays the proportions of illusion type responses given in each condition, which necessarily add up to 1. Straight responses were predominant in the control conditions, whereas a larger proportion of slalom responses were given in the tilted conditions. At mid and high eccentricities for the tilted lines, squirm responses were most common, whereas at low eccentricity nearly half of the responses for the tilted condition were given as straight. That is, the proportion of slalom responses does not increase with eccentricity in the tilted conditions. Instead, straight and – to a lesser degree – slalom responses are being substituted with squirm responses.

In addition, the dual response paradigm of this experiment allows an analysis of the perceived amplitude on specifically those trials where a tilted display was shown, and a slalom illusion was perceived. This gives better insight into whether eccentricity affects the amplitude of the slalom illusion, as it allows to discriminate between a situation where the mean amplitudes displayed in Fig. 8 increased only because fewer straight and more squirm responses were given, and a situation where the perceived amplitude of the slalom illusion itself was affected by retinal eccentricity. For trials at low eccentricity, a mean amplitude response of 5.42 pixels was observed (N = 15, SE = 0.88). At mid eccentricity, this was 7.16 pixels (N = 17, SE = 1.12), and at high eccentricity, it was 7.63 pixels (N = 20, SE = 1.05). This constitutes a monotonic increase of the amplitude with eccentricity. As a consequence of filtering the data by the reported illusion type, not all participants had data points in all conditions, and therefore not all participants could be taken into an inferential analysis. Six participants had to be excluded, and no effect of eccentricity on the magnitude of the slalom illusion was found in a repeated measures ANOVA with Greenhouse - Geisser correction [F(1.25, 16.2) = 1.13, *p* = 0.06].

The current results are a refutation of the alternate hypothesis that the slalom illusion would not occur under conditions of constant fixation. Even in the absence of eye movements, more slalom responses and greater perceived amplitudes were reported for tilted line displays than for vertical line control displays. This implies that the slalom illusion can result both from a trajectory that is projected across the retina, such as in the current experiment, and a trajectory that is stabilised on the retina through a smooth pursuit viewing strategy, as in all previous experiments. In addition, further evidence is provided for the existence of the Squirming illusion^21^, a variant of the Furrow illusion^18,19^, both of which occur outside of central vision and are similar to the Bicycle illusion observed in small, low-contrast displays. The slalom illusion was not replaced completely at mid and high eccentricities, but the frequent occurrence of the squirming illusion interfered with the statistical assessment of eccentricity effects on the magnitude of the slalom illusion, leaving only a preliminary conclusion that neither the occurrence, nor the magnitude of the slalom illusion were affected by the eccentricity of the stimulus display.

## Discussion

The slalom illusion as originally reported by Cesàro and Agostini^1^ was repeatedly replicated over the course of four experiments. In the current study, the magnitude of the slalom illusion was shown to increase through partial occlusion and decrease again with shorter ISIs of occlusion. The slalom illusion was still observed under conditions of constant fixation, with a greater reported amplitude outside of central vision. However, it could statistically not be shown that this effect was driven by the slalom, rather than the qualitatively distinct squirming illusion. An inverted stimulus display, where a sinusoidal trajectory intersected with vertical lines, did not induce the expected illusory underestimation of the trajectory magnitude. These findings define new empirical boundaries for the slalom illusion.

The current results agree with the view of Cesàro and Agostini, whereby the slalom illusion occurs as a compromise between local distortions towards perpendicular angles and the veridical horizontal motion direction. A potential explanatory mechanism for the ineffectiveness of the inverse slalom illusion is proposed to be *adaptation*. The visual system has evolved to represent transients in stimuli, and constant stimulation leads to perceptual fading and opposite after-effects due to neuronal recalibration^51–54^. In the context of the slalom illusion, it appears unlikely that this neuronal recalibration would occur so quickly, as this commonly requires several seconds of stimulation^55^. However, a number of single-cell studies on direction-sensitive motion cells in macaque MT cortex have also demonstrated the existence of short-term adaptation within tens of milliseconds^56–58^. These cells appear to exhibit an inherently biphasic response, whereby a strong initial response to their preferred direction is quickly followed by a lowered firing rate and a stronger response to non-preferred directions, to the same effect as long-term neuronal adaptation. Because the motion signal in the inverted slalom display is not constant in direction, such short-term adaptation would not occur as it would in the original slalom display, and the stronger motion signal would dominate or overrule the local distortions more easily. The original slalom illusion is then fundamentally caused by a combination of the visual system’s preference for perpendicular angles *and* its preference for transient motion directions over constant motion directions. Further experimental research would need to be conducted to further substantiate this theoretical view; in particular, the influence of short-term adaptation effects could be tested in psychophysical experiments by systematically manipulating the variation in motion direction prior to the moment of intersection.

In addition, we propose that the ‘perceptual compromise’ explanation put forward by Cesàro and Agostini is not a passive, bottom-up process of summing motion vectors in a spatiotemporal integration window, but rather a post-hoc interpretation of the trajectory in the light of the available evidence. Principally, the observation that faster speeds during occlusion lead to smaller estimated amplitudes would suggest that amplitude estimation is only made *after* the re-appearance of the moving dot. As mentioned above, similar effects have been shown in the case of curved paths for apparent motion^34,35^.

In summary of this theoretical view, the weight of the perceptual evidence for the veridical horizontal trajectory is proposed to be especially weak in the case of rapidly adapted constant motion direction, partial occlusion, and strong distortions around the points of intersection. In these situations, the slalom illusion was indeed shown to have the largest magnitude.

## Conclusion

The motion path of an object is more than a contiguous sequence of position and motion direction signals. In the slalom illusion, a number of circumstances are brought together that demonstrate this clearly, by giving temporary local biases of motion direction strong leverage over the perceived trajectory. The present study has outlined the main empirical boundaries of the phenomenon (constant direction of the veridical motion, and either smooth pursuit or near-central fixational viewing conditions) and has emphasised the interpreted and balanced nature of the compromise made between visual input and perceptual biases.

## Methods

### Ethical approval and informed consent

The project received ethical approval from the Research Ethics Committee of the Development & Society Faculty at Sheffield Hallam University, in accordance with the Declaration of Helsinki. All participants gave their written informed consent.

### Participants

Participants were recruited through Sheffield Hallam University’s Psychology credit scheme as well as through opportunity sampling from the general student population. Participants were naive as to the phenomenon investigated. Inclusion criteria were having normal or corrected-to-normal vision and being at least 18 years old.

### Design

For experiments 1-3, repeated-measures designs were employed, with one independent variable, *background*. The dependent variable was the amplitude of the trajectory, given by the height of the response line, measured in pixels.

### Apparatus

All experiments were programmed in Psychtoolbox-3 for MATLAB^59^. Experiments 1-3 were presented on a NEC MultiSync FP2141sb 22” CRT monitor with a viewable area of 406 ×304.6 mm. The experiments were run with a spatial resolution of 1600 ×1200 pixels and a temporal resolution of 85 Hz. For Experiment 4, a Tobii Pro T120XL eye tracker was used to record eye movements and stimuli were presented on the Tobii Pro T120XL incorporated monitor with a resolution of 1920 ×1200 pixels.

### Stimuli

The experimental display consisted of two components: The *background*, meaning the static stimuli, and the *moving dot*, which moved across the background. The dot was black and had a diameter of 2 mm. The screen was white (luminance 255).

### General procedure

Before starting the experimental task, participants were assisted in a trial run consisting of ten repetitions of random stimuli from all the relevant experimental conditions. A chin rest was used, placed at 60 cm from the monitor. The task consisted of following the dot, which moved back and forth continuously across the background, and estimating the vertical displacement of the trajectory. To input their responses, participants adjusted the height of a probe line, placed at the centre-bottom of the display and present on the screen concomitantly with the stimuli. The response line had a starting height randomly assigned from 1 to 20 pixels and was adjustable through the use of ‘up’ and ‘down’ arrow keys. Pressing the space bar confirmed the response and the next trial commenced immediately. There were ten repetitions per experimental condition. Participants completed the experimental task at their own pace, and the duration was 30 minutes on average, excluding the initial instructions and practice trials.

### Experiment 1 - Occlusion

#### Stimuli

The dot moved across the monitor at a speed of 5 cm/sec. The angle of intersection for all the tilted lines was 41.4°. In the three triangle conditions the background consisted of 7 isosceles triangles, with a base of 4.5 cm and two equal edges (the tilted lines) of 3 cm. The triangles were placed at 1 cm distance from each other at the base. The occluding triangles (Fig. 2B) were black and hid the trajectory of the dot when it intersected their surface. The gray triangles (Fig. 2C) had a mid-gray luminance of 127 RGB and did not occlude the trajectory of the dot, as the black dot crossed in front of them. The transparent triangles (Fig. 2D) had a black contour of 1 mm, but were not filled, and the trajectory of the dot was visible when it translated them. The original slalom condition (Fig. 2A) consists of a series of seven modules of two tilted lines corresponding to the edges of the triangles from the triangles conditions; the distance between the modules is 1 cm and the tilted lines are 1 mm thick and 2.5 cm long. The vertical lines in the control condition (Fig. 2E) were black, 1 mm thick and 2 cm long. The centre of the experimental display coincided with the centre of the screen in all conditions.

#### Data analysis

Thirty participants ran 50 trials each, in random order. Two outliers were removed (z > 3.29). To achieve a normal distribution, the data were log-transformed. A repeated-measures one-way univariate ANOVA was performed.

### Experiment 2 - ISI

#### Stimuli

All backgrounds to the displays were taken from Experiment 1. The non-occluded condition was identical to the original slalom condition (Fig. 2A), whereas the occluded backgrounds were identical to black triangles (Fig. 2B). The speed of the moving dot in the *visible* parts of the trajectory was 5 cm/s in all conditions. In conditions featuring black occluding triangles, the speed in the occluded parts of the trajectory was either kept constant (*original ISI* condition of 470 ms), increased by 100% (*short ISI* condition of 235 ms; Fig. 2C), or decreased by 33.33% (*long ISI* condition of 705 ms; Fig. 2D).

#### Data analysis

Seventeen participants ran 40 trials each. There were no outliers, and the data was not transformed prior to performing a repeated measures one-way univariate ANOVA.

### Experiment 3 – Inverted

#### Stimuli

The dot moved along the sinusoidal trajectory at a speed of 6.5 cm/s, corresponding to a 5 cm/s horizontal speed. The trajectory of the dot was sinusoidal, with an amplitude of 10 pixels, so that it was within a similar order of magnitude as the perceived illusory amplitudes. The trajectory had a phase of 0 (meaning that it starts in the middle of the vertical range of the trajectory), and a frequency of 7 cycles across the screen. The background for the moving dot depends on the experimental condition, as follows: in the *inverted slalom* condition, there are 7 modules each of two vertical lines (Fig. 6A), in the *inverted occluded* condition, there are 7 black squares (Fig. 6B) in the *inverted control* condition there are 7 modules of lines tilted at 41.4° (Fig. 6C), and in the *blank* condition the dot moves across a white, blank, background (Fig. 6D).

All lines, including the vertical edges of the squares, were placed at the points where sin(x) = 0, leading to the following angles of intersection: in the *inverted slalom* and *inverted occluded* conditions, the angle of intersection was 70.7° (and bias towards perpendicularity would decrease the illusion), whereas in the *inverted control*, the angle of intersection was 59.3° (and bias towards perpendicularity would increase the illusion). The centre of the experimental display coincided with the centre of the screen in all conditions.

#### Data analysis

Forty participants ran 40 trials each. There were no outliers, and the data was not transformed prior to performing a repeated measures one-way univariate ANOVA.

### Experiment 4 – Retinal eccentricity

#### Stimuli

The stimulus displays consisted of four inducing lines (tilted or vertical, depending on condition), forming two modules. The distance between the two lines which make up a module was 1.86 cm at the base, whereas the distance between the two modules was 0.93 cm. At this size, the stimulus covered the maximum display area that would fit within central vision (5° of visual angle). For consistency, the same size was maintained for the stimuli presented in the peripheral condition. The angle of intersection with the dot trajectory was 41.4° for all tilted lines, whereas for the control vertical lines the angle of intersection was 90°. In all six conditions the moving dot now had a diameter of 5 pixels (1.25 mm), and traversed the two modules twice, always from left to right. The position of the stimuli on the screen depended on the condition as described below. In the *low eccentricity* conditions, the modules were aligned vertically with the fixation point, and were placed either directly on top or below the fixation point; this ensured both that the fixation point was not incorporated in the stimuli, and that the modules were in their entirety within the 5° central vision field. In the *mid eccentricity* conditions, the modules were aligned horizontally with the fixation point, and were placed either to the left, or to the right of it, always at a 8.4° visual angle. In the *high eccentricity* conditions, the modules were aligned horizontally with the fixation point, and were placed either to the left or to the right of it, always at a 16.8° visual angle.

#### Procedure

The experimental task consisted of 108 trials in random order (18 repetitions per condition). Twenty participants were asked to look at the black dot situated in the centre of the screen and to press Enter while they are fixating the black dot. If they were fixating, the dot changed its colour to green, indicating that they can proceed with pressing the Enter key. After pressing Enter, the fixation point remained present on the screen and the stimuli appeared, consisting of the dot crossing the module of lines twice, always in the same direction (left to right). Following that, the stimuli disappeared and were replaced by the adjustment line used to measure the magnitude of the illusion, as in previous experiments. After adjusting the line participants had to press the Space bar and this led to the final part of the trial, where they were offered a forced choice between three descriptions of the dot trajectory: ‘straight’, ‘slalom’ and ‘squirm’. Once this answer was given by using the corresponding key (1, 2, and 3, respectively), the following trial commenced immediately.

#### Data analysis

One of the participants completed only 90 trials, whereas the remaining 19 participants each completed all 108 trials. In total, data for 2142 trials were collected over 20 participants. One trial was removed because no valid eye movement measurements were recorded. Then, trials where the gaze was at the indicated fixation position for less than 80% of the trial time were removed. 1831 trials across 20 participants remained in the data set.

The amplitude response data were log-transformed, in order to obtain a symmetrical distribution. A repeated-measures two-way univariate ANOVA was performed. The first independent variable, *stimulus type*, had two levels: *tilted lines* and *vertical lines*. The second independent variable, *eccentricity* had three levels: *low eccentricity* (within 5°), *mid eccentricity* (8.4°), and *high eccentricity* (16.8°). The dependent variable of the ANOVA, *amplitude*, is continuous and operationalised as the height of the response line measured in pixels.

## Data Availability

The data that support the findings of this study are openly available on Open Science Framework at 10.17605/OSF.IO/QXY7P.
